# Understanding the Engineering Tactics to Achieve the Stabilized Anode in Next‐Generation Zn‐Air Batteries

**DOI:** 10.1002/EXP.20240054

**Published:** 2025-03-02

**Authors:** Subramani Surendran, Yoongu Lim, Seona Lee, Sebastian Cyril Jesudass, Gnanaprakasam Janani, Heechae Choi, Gibum Kwon, Kyoungsuk Jin, Uk Sim

**Affiliations:** ^1^ Hydrogen Energy Technology Laboratory Korea Institute of Energy Technology (KENTECH) Jeollanamdo Republic of Korea; ^2^ Department of Materials Science and Engineering Chonnam National University Gwangju Republic of Korea; ^3^ Department of Chemistry Xi'an Jiaotong‐Liverpool University Suzhou China; ^4^ Department of Mechanical Engineering University of Kansas Lawrence Kansas USA; ^5^ Department of Chemistry Korea University Seoul Republic of Korea; ^6^ Research Institute NEEL Sciences, INC Jeollanamdo Republic of Korea; ^7^ College of Chemistry and Chemical Engineering, Henan Key Laboratory of Function‐Oriented Porous Materials Luoyang Normal University Luoyang PR China

**Keywords:** anode stabilization, electrolyte, energy storage, surface engineering strategy, zinc‐air battery

## Abstract

The modern technical era demands sustainable and green energy production and storage methods that overcome the limitations of conventional fuel resources. Electrochemical energy storage (ECS) technologies are widely anticipated to store and release energy on repeated cycles for domestic and commercial utilization. Several ECS devices were developed over the years to achieve higher energy density and energy sustainability. Zn‐air batteries are developed to deliver higher energy density and their lower maintenance, flexibility, and rechargeability made them the significant sustainable energy device. However, the Zn anodes face several issues due to dendrite formation during several discharge cycles, HER at higher negative potentials, and corrosion behavior. Therefore, Zn‐anode design strategies and significant electrolyte modifications were adopted to limit the critical issues. The review promptly exhibits the significance of Zn‐air battery and their construction strategies. The present review highlights the rational design strategies for the stabilization of the Zn anode, such as coating with a passive layer, heterostructure and alloy‐composite formation, and the major electrolyte modifications, such as using organic electrolytes, additives in aqueous electrolytes, and solid‐state polymer gel electrolytes. The review is expected to attract a wide range of readers, from beginners to industrialists, which serve as a guide for developing Zn‐air batteries.

## Graphical abstract

This review highlights crucial design strategies for zinc anodes and significant electrolyte modifications to address key challenges in zinc‐air battery development. The article is intended to serve as a valuable resource for a broad audience, from novice researchers to industry professionals, guiding the development of Zn‐air batteries.

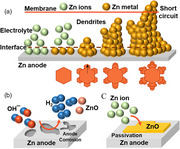

## 1 Introduction

Rapid industrialization has urged the need for green and sustainable energy resources and more efficient energy storage technologies than conventional fuel resources. Renewable energy technologies are crucially developed to cater the energy demands and promote the green energy transition [1, 2, 3]. Energy storage devices like batteries, supercapacitors, and fuel cells have been widely used and upgraded over the years to achieve higher energy storage efficiency.

Notably electrochemical energy involving heterogeneous redox reactions was considered as the future scope of green and reliable energy technology. Portable energy storage technologies like batteries and supercapacitors, which operate via electrochemical redox reactions, enable us to preserve energy from various sources and provide several rechargeable cycles. Besides, several domestic and commercial sectors, like households, electric vehicles, and industries, majorly depend on batteries as the primary energy reserves to retain power during power interruption from the grid source [4, 5]. Hence, the development and advancements of batteries prove to be highly valuable to achieving energy sustainability, reducing pollution caused by conventional fuels, and promoting the green energy transition.

**FIGURE 1:**
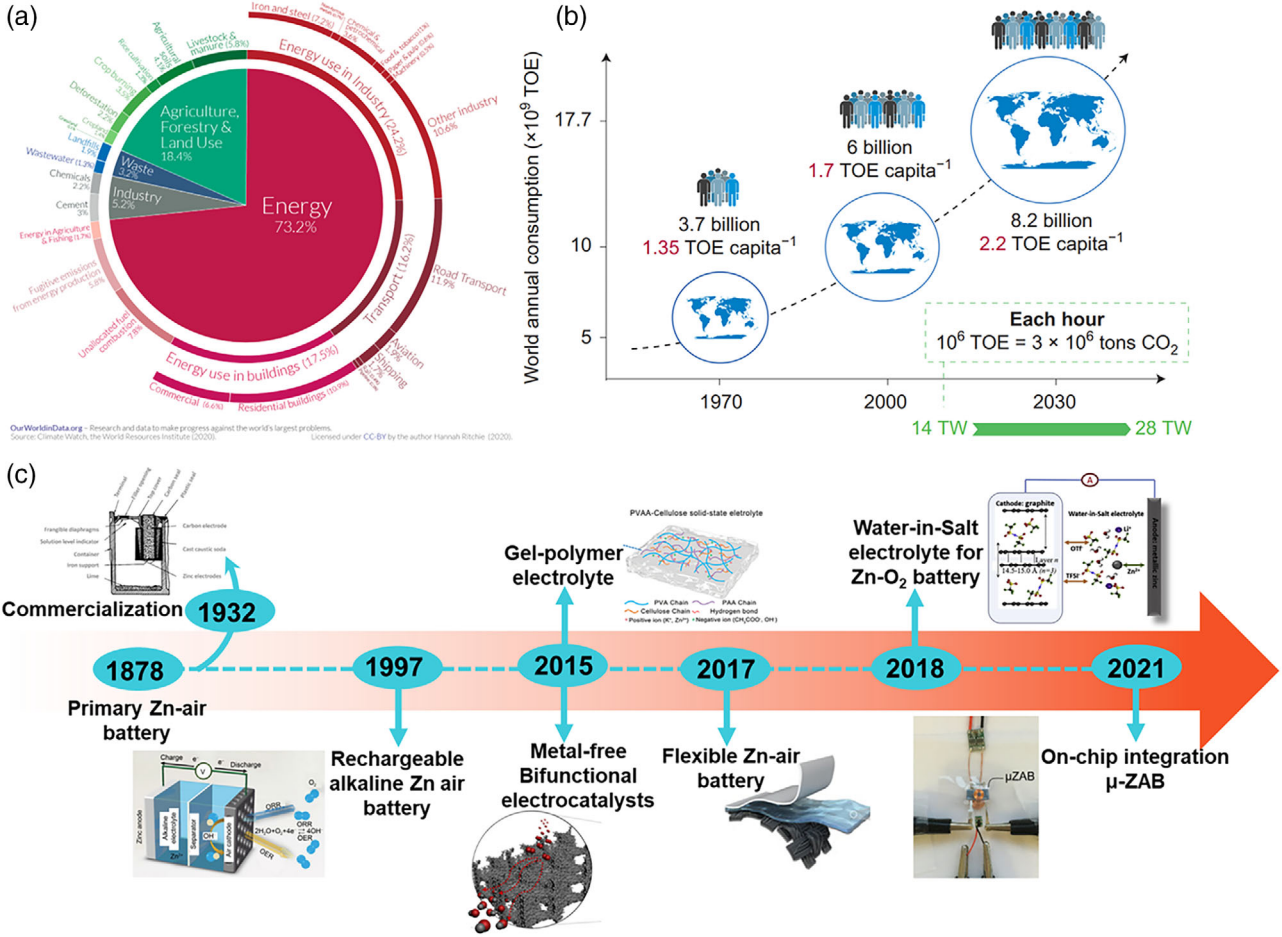
(a) Representation of global CO_2_ emissions from various sectors [6]. (b) Representation of global per capita energy consumption. Reproduced with permission [7]. Copyright 2015, Elsevier. (c) Timeline of Zn‐air battery development. Reproduced with permission [13, 14, 15, 16, 17, 18, 19]. Copyright, Royal Society of Chemistry, American Chemical Society, and Wiley‐VCH.

Figure 1a depicts the global CO_2_ emissions from multiple sectors, and the energy sector has the highest CO_2_ emissions at 73.2% [6]. Hence, it is imperative to reduce global emissions by increasing the reliability of electrochemical energy storage technologies. Global per capita energy consumption increases rapidly every year, along with the ever‐increasing population and global CO_2_ emission (Figure 1b) [7]. Electrochemical energy storage methods also offer multiple benefits compared to other energy storage forms. For instance, batteries are light in weight and compact, making them well‐adapted for portable applications [8, 9]. The utilization of electric vehicles is increasing due to the sustainable progress toward green energy, which demands the development of energy storage. The driving range of electric vehicles depends on the performance of batteries; hence, it is imperative to achieve higher energy density [10, 11, 12].

Furthermore, electrochemical storage systems can quickly charge and discharge, making them fit for various uses requiring brief energy releases. During this transitional period, as we move toward more eco‐friendly energy solutions, technologies able to chemically store and release electrical power will become ever more essential [20]. By allowing us to save power generated from natural renewables and transform it back to electricity conveniently when required, these innovations will aid our efforts to lessen dependence on carbon emissions and help counter the damaging consequences of a changing climate [21].

Zinc‐air batteries are rechargeable batteries that use zinc as an anode and oxygen reactions at the cathode to supply energy. Zinc‐air batteries can store much power for their size, cost little to make, and are eco‐friendly [22, 23]. Even though multiple components are involved in the zinc‐air battery system, an essential part relies on the Zn‐anode, which must be designed carefully to generate an effectual performance and long cycle life [24, 25, 26, 27, 28]. Over several decades, Zn‐air batteries have evolved from primary to solid‐state/flexible rechargeable batteries and micro‐batteries (Figure 1c) [13, 14, 15, 16, 17, 18, 19].

Despite Zn‐air batteries, Li‐air, Na‐air, and Al‐air batteries have adopted several anode stabilization strategies that limit the formation of dendrites, enhance corrosion resistance, avoid electrode passivation, and suppress HER. For instance, Li‐air batteries are unstable in an oxygen environment where side reactions form intermediates like LiO_2_, O_2_^−^, O_2_^2−^, and O_2_ by electrolyte decomposition, forming a passivating layer on the electrode surface [29, 30, 31]. Therefore, researchers proposed the use of organic electrolytes like dimethyl sulfoxide (DMSO) [32], tetraethylene glycol dimethyl ether (TEGDME) [33], tetramethylene sulfone (TMS) [34], *N*,*N*‐dimethylacetamide (DMA) [35], lithium bis(trifluoromethane sulphonyl) imide (LiTFSI) with LiNO_3_ [36], propylene carbonate (PC) [37] and many other electrolytes, which demonstrated high discharge plateau and higher specific capacity. However, each had its disadvantages of low Li salt solubility, toxicity, safety, volatility, decomposition at higher voltages, instability toward oxide intermediates, and thermal instability. Besides, the Li‐air battery was designed as an all‐solid‐state device that actively limits dendrite formation as the non‐uniform deposition is effectively controlled.

Similarly, the Na anode faces dendrite formation, HER, and the passivation layer formed due to H_2_O/CO_2_ crossover forming NaOH/Na_2_CO_3_, and corrosion due to peroxide and superoxide crossover effects [38, 39]. The anode stabilization of Na‐air batteries focuses on maintaining a stable solid‐electrolyte interface (SEI), which has high ionic conductivity, low electrical conductivity, small thickness, and flexibility to suppress Na dendrites mechanistically. Anode design strategies like forming a protective layer of NaF [40], Na‐Li alloys [41], and solid‐state Na‐air battery [42] to enhance the efficiency of the Na‐air battery. In addition, several electrolyte modifications were adopted with organic electrolytes [43], inorganic salts [44], and gel electrolytes [45] to stabilize the Zn anode, however, decomposition at higher voltages, volatility, low salt solubility, and lower ionic conductivity were observed. Hence, developing metal‐air batteries requires critical assessment and choosing appropriate elements to construct better energy storage technologies.

Among the different design strategies for a Zn‐anode, the anode's surface area is a crucial criterion. The increase in the surface area that touches air and the electrolyte helps the chemical reaction generate more current faster. This instigates the trend of making anodes with large surface areas inside small battery sizes [24, 25]. Similarly, incorporating carbon‐based substances, like carbon nanotubes or graphene, with a substantial surface area and electrical conductivity can vastly boost the performance of the Zn‐anode [25] Another strategy comprises enveloping customary metal anodes, like stainless steel, with activating substances that amplify the anode's reactivity [46]. The innovation and refinement of anode blueprint for zinc‐air batteries bear momentous consequences for the progression of this technology. Enhancing the efficiency and resoluteness of the anode makes it reliable for amplifying the energy density and lifespan of the battery, rendering it more attractive for an array of applications like electric vehicles, grid storage, and portable electronic devices.

The present review includes the significance and advancement of Zn‐air batteries for modern energy storage technologies. It emphasizes the problems and challenges faced by Zn‐air battery anodes and their possible solutions. The review clearly explains various strategies in designing Zn anode and electrolyte to eliminate the issues faced by the Zn anode during charge–discharge cycles.

## 2 Mechanism of Zn‐Air Battery

**FIGURE 2:**
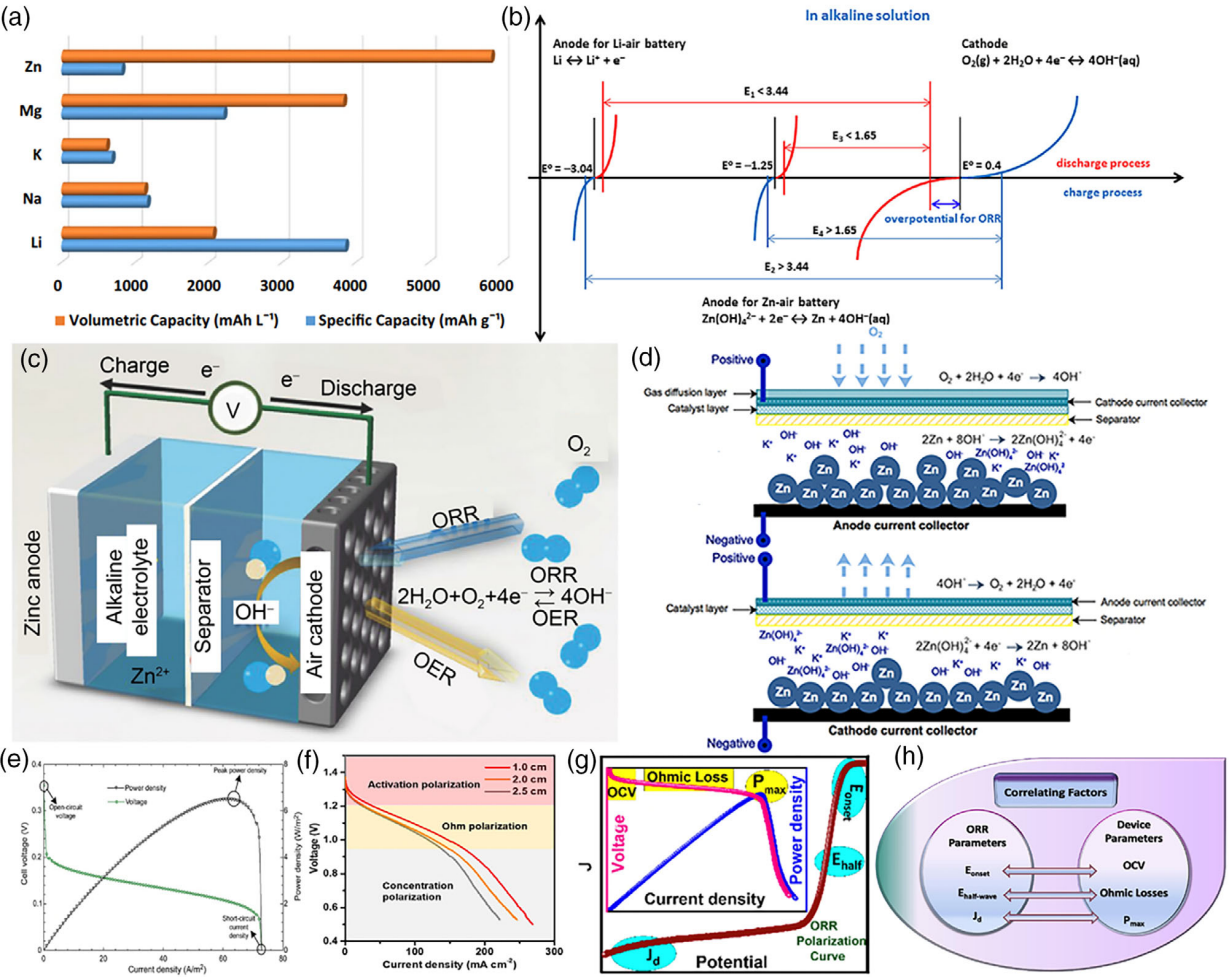
(a) Comparison of volumetric capacity and specific capacity in various metal‐air batteries [47]. (b) Standard potential and overpotential curves for Zn‐air battery reactions. Reproduced with permission [50]. Copyright 2011, Wiley‐VCH. (c) Representation of Zn‐air battery [14]. (d) Mechanism of discharging (1) and charging (2) reactions in Zn‐air battery [51]. (e) Polarization and power density curves. Reproduced with permission [52]. Copyright 2020, Elsevier. (f) Contribution to polarization losses in Zn‐air battery. Reproduced with permission [53]. Copyright 2020, Elsevier. (g) ORR curves and polarization‐power density curves of Zn‐air battery. (h) Relation between parameters of ORR and Zn‐air battery. Reproduced with permission [54]. Copyright 2021, American Chemical Society.

The metal redox potential and the cathodic electrocatalytic reaction determine the theoretical capacity of the metal‐air batteries. Zn‐based batteries reveal promising energy storage performance compared to Li, K, Na, and Mg‐based batteries (Figure 2a) [47]. Zn‐based batteries exhibit a relatively high volumetric capacity, about 5855 mAh L^−1^, with a considerable specific capacity of about 820 mAh g^−1^ and a theoretical specific energy density of 1080 Wh kg^−1^ [48]. However, a low specific capacity for Zn‐air battery is observed compared to other metal‐air batteries due to the lower redox potential of −0.76 V for Zn/Zn^2+^ than Li/Li^+^ (−3.04 V), Mg/Mg^2+^ (−2.36 V), and Na/Na^+^ (−2.71 V). Despite the lower specific capacity, Zn‐based batteries can be handled at ambient conditions, are low‐cost, low maintenance, higher abundance, and have improved rechargeability, which exhibits higher potential as energy storage devices. The alkaline Zn‐air battery consists of an anode, air cathode, and alkaline electrolyte, where the air cathode performs ORR during discharge and OER during the charging process [26, 49].

The simplified half‐cell reactions for discharge/charge reactions in Zn‐air batteries are explained below.

$$\text{Anode:}\;\mathrm{Zn}+4\;{\mathrm{OH}}^{-}↔\mathrm{Zn}{\left(\mathrm{OH}\right)}_{4}^{2-}+2e^{-}\left(E_{\mathrm{an}}∼-1.25\;V_{\mathrm{SHE}}\right)$$

$$\text{Cathode:}\;O_{2}+2H_{2}O+4e^{-}↔4{\mathrm{OH}}^{-}\left(E_{\mathrm{cath}}∼0.4\;V_{\mathrm{SHE}}\right)$$

$$\text{Overall:}\;2\mathrm{Zn}+O_{2}\to 2\mathrm{ZnO}\left(E^{0}=1.65\;V\right)$$

The ORR and OER processes at the air cathode should overcome the overpotential, which is an additional required potential beyond the theoretical voltage (Figure 2b) [50]. The Zn‐air battery generates a lower theoretical voltage of 1.65 V compared to the Li‐air battery (2.91 V). The construction of a Zn‐air battery consists of anode and cathode well isolated by a separator and filled with anolyte and catholyte, respectively (Figure 2c). The air cathode of the constructed Zn‐air battery performs ORR during discharge, consuming O_2_ and OER during the charging cycle with O_2_ evolution via four electron transfer cycle. During discharge, the Zn metal at the anode is oxidized to Zn(OH)_4_^2−^ by donating electrons to the circuit, and the cathode reduces O_2_ through the ORR process by accepting electrons from the circuit (Figure 2d (1)) [51]. Hence, a current flow in the external circuit through load is achieved. During charging, the electrons are supplied from externally applied voltage, which reduces the Zn(OH)_4_^2−^ ions into Zn metal at the anode by accepting electrons, and the OH^−^ ions at the cathode are converted to O_2_ by the OER process (Figure 2d (2)). Therefore, rechargeability of the Zn‐air battery can be achieved feasibly through ORR and OER processes at the cathode, which is facilitated by the electrocatalyst.

The open circuit voltage (OCV), voltage discharge plateau, and the peak power density in the polarization‐power density plots for the fabricated Zn‐air battery are essential attributes to determine its performance (Figure 2e) [52]. The theoretical output voltage of the Zn‐air battery is always decreased (<1.65 V) due to overpotentials. The overpotential is contributed by various factors like ohmic overpotential, activation overpotential, and concentration overpotential (Figure 2f) [53].

The polarization curves of the Zn‐air battery can be explained by the following equation:[53]

$$E=E_{\mathrm{theoretical}}-\frac{RT}{\alpha F}ln\left(\frac{i}{i_{0}}\right)-\frac{RT}{nF}ln\left(\frac{i_{L}}{i_{L}-i}\right)-iR_{i}$$

where *n*, α, *R*, *T*, *F* represent number of electrons transfer, electron transfer coefficient, gas constant, temperature, and Faraday constant (96,485 C mol^−1^). Also, the *i*, *i_0_*, *i*_L_, *E*, and *E*_theoretical_ represent the observed current density, exchange current density, limiting current density, electrode potential, and theoretical open circuit voltage, respectively. The terms in the polarization equation $iR_{i}$, $\frac{RT}{\alpha F}ln\left(\frac{i}{i_{0}}\right)$, and $\frac{RT}{nF}ln\left(\frac{i_{L}}{i_{L}-i}\right)$ correspond to ohmic overpotential, activation overpotential, and concentration overpotential, which reduces the actual output voltage to less than the theoretical output voltage (1.65 V).

The performances of a Zn‐air battery highly depend on the ORR performances of the electrocatalyst, such as the onset potential (*E*_onset_), half‐wave potential (*E*_1/2_), and limiting current density (*i*_L_) of the air cathode, which determine the onset voltage and power density of the Zn‐air battery (Figure 2g) [54]. The ORR performance of the electrocatalyst correlates strongly with the performance of the Zn‐air battery where the *E*_onset_ of the ORR electrocatalyst relates to open circuit voltage, *E*_1/2_ to ohmic losses, and *i*_L_ to peak power density (*P*_max_), respectively (Figure 2h).

## 3 Zn Anode Challenges

Despite the importance of developing cathode materials, the Zn anode in rechargeable Zn‐air batteries also faces critical challenges, like dendrite formation, corrosion, HER, and electrode passivation, which degrade the charge storage performances. Therefore, studies have been devoted to understanding the degradation phenomena and remedies to achieve efficiency in energy storage.

### 3.1 Dendrite

**FIGURE 3:**
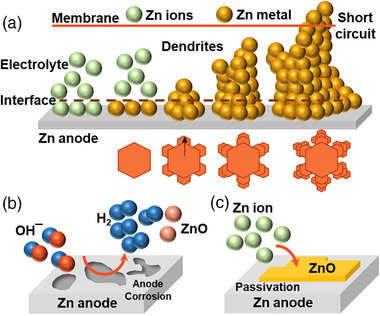
(a) Evolution of dendrites on Zn anode, (b) anode corrosion and HER dominant reactions, and (c) passivation of the Zn anode.

During discharge–charge cycles, the Zn anode constantly forms Zn ions and again deposits Zn metal (Figure 3a). The Zn metal deposition from the Zn(OH)_4_^−^ ions during charging cycles gradually forms a non‐uniform deposition and accumulates at specific sites on the Zn electrode surface. After several charge–discharge cycles, the Zn deposition builds up and forms a needle‐like morphology. The continuous growth of the Zn dendrites reaches and pierces the separator leading to the short‐circuit and failure of the battery. The formation of dendrites is controlled by the mass transfer process of electrolytes, current density distributions, and the concentration of hydroxide and Zn(OH)_4_^2−^ ions. The Zn deposition can be actively controlled during cycling by operating current density and electrode overpotential and by necessary electrode and electrolyte modifications, as discussed in the later section [55, 56, 57].

### 3.2 Corrosion and HER

The harsh negative potentials of the Zn‐air battery led to Zn anode corrosion and caused H_2_ evolution (HER) (Figure 3b). Since the standard reduction potential of HER is practically higher than ZnO/Zn, HER occurs inevitably during rechargeable Zn‐air battery cycles [58]. When HER occurs, the internal pressure of the Zn‐air battery increases, resulting in swelling and a shortened life span. Besides, the unwanted side reactions can lead to capacity decay and decreased efficiency of the Zn‐electrode. Apparently, under alkali conditions and at higher negative potentials, the Zn‐electrode decays, and HER also takes place inevitably.

$$\text{Zn corrosion: Zn}+2{\text{OH}}^{-}\to \text{ZnO}+H_{2}O+2e^{-}\left(-1.26\;V_{\text{SHE}}\right)$$

$$\text{Anodic HER:}\;\mathrm{Zn}+2H_{2}O\to \mathrm{Zn}{\left(\mathrm{OH}\right)}_{2}+H_{2}$$

$$H_{2}\;\text{evolution:}\;2\;H_{2}O+2e^{-}\to 2{\text{OH}}^{-}+H_{2}\left(-0.83\;V_{\text{SHE}}\right)$$

### 3.3 Passivation

Passivation occurs at the Zn electrode when ZnO is supersaturated in the alkaline electrolytes and deposited on the electrode surface, forming an insulating layer for redox reactions during discharge (Figure 3c) [59, 60]. The insulating layer blocks the surface from electrolytic ions during discharge, affecting the specific capacity of the Zn‐air battery and limiting its rechargeability and life span. Liu et al. observed that the total time of Zn to passivate in the electrolyte is equal to the sum of the saturating time of Zn(OH)_4_^2^¯, the time of porous ZnO layer formation, and the time for dense ZnO layer formation. Besides, the study suggests that at high current density, a dense ZnO layer forms, which passivates the electrode, whereas at low current density, porous ZnO forms, reducing the passivation of the electrode. However, insights into the study of passivation are an active research area that helps us understand the effects of electrode degradation.

## 4 Zn Anode Modifications

**FIGURE 4:**
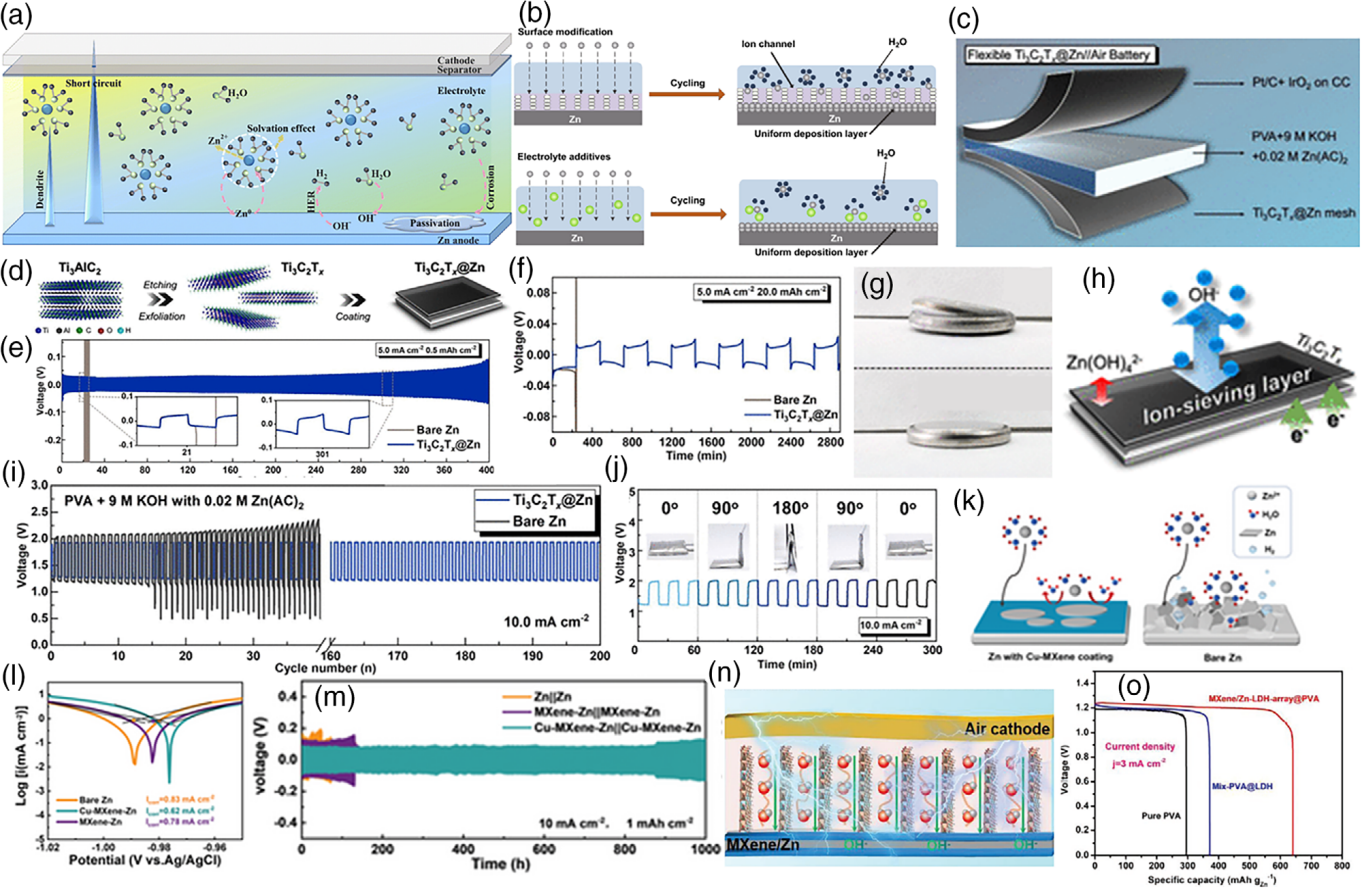
(a) Representation of issues in Zn anode. Reproduced with permission [61]. Copyright 2022, Royal Society of Chemistry. (b) Remedies to suppress dendrite formation by Zn surface and electrolyte modification. Reproduced with permission [62]. Copyright 2021, Springer Nature. (c) Representation of flexible Zn‐air battery with Ti_3_C_2_T*_x_* @Zn anode. (d) Preparation scheme of Ti_3_C_2_T*_x_* @Zn anode. (e) Charge–discharge performances of ZAB with Ti_3_C_2_T*_x_* @Zn anode. (f) Charge–discharge performances at 5 mA cm^−2^ and 20 mAh cm^−2^. (g) Optical images of ZAB with bare Zn and Ti_3_C_2_T*_x_* @Zn anode. (h) Representation of ZAB performances at Ti_3_C_2_T*_x_* @Zn anode, (i) charge–discharge performances, and (j) bending tests of flexible ZAB with Ti_3_C_2_T*_x_* @Zn anode, respectively. Reproduced with permission [63]. Copyright 2023, American Chemical Society. (k) Representation of ZAB performances with Cu‐MXene‐Zn anode, (l) polarization curves of ZAB with Cu‐MXene‐Zn anode, (m) charge–discharge performances. Reproduced with permission [64]. Copyright 2023, Wiley‐VCH, and (n) representation of ZAB performances in MXene/Zn anode. (o) Polarization curves of MXene/Zn‐LDH‐array@PVA electrode for ZAB. Reproduced with permission [65]. Copyright 2022, Wiley‐VCH.

Zinc (Zn) anodes suffer from several problems that limit their performance and lifetime in batteries, such as dendrite growth, corrosion, HER, and electrode passivation. Various strategies can be used to counteract these issues (Figure 4a) [61]. An effective strategy is to coat the Zn anode with an insulating material such as carbon or polymer, which reduces the rate of dendrite growth and prevents the shuttle effect (Figure 4b) [62]. The surface modification strategy includes forming a heterostructure of Zn with MXene support, coating the Zn electrode with a polymer, forming a ZnF_2_ layer over the Zn electrode, and forming a porous Zn anode. Another approach is to control the electrolyte content, which selectively suppresses undesired by‐products and dendrite growth. Using these techniques, the performance and lifetime of the Zn anode can be greatly improved, providing improved battery performance.

Yang et al. [63] designed the rechargeable aqueous flexible Zn‐air battery with Ti_3_C_2_T*_x_* @Zn metal, where the MXene layer acts as a protective layer (Figure 4c,d). The Ti_3_C_2_T*_x_* @Zn metal anode delivers better cycle stability than the bare Zn anode, as shown in Figure 4e. The bare Zn anode delivered cycling stability of less than 25 cycles whereas Ti_3_C_2_T*_x_* @Zn metal anode had better stability for 400 h. Besides, the fabricated coin cell with Ti_3_C_2_T*_x_* @Zn anode and IrO_2_ cathode shows better durability for 2800 min at 5 mA cm^−2^ current density and 20 mAh cm^−2^ capacity than performed with bare Zn anode (Figure 4f). Notably, the swelling was observed in the fabricated coin cell with bare Zn anode after the cycling test, where the coin cell with Ti_3_C_2_T*_x_* @Zn anode displayed relatively higher durability (Figure 4g).

Besides, the fabricated flexible Zn‐air battery with PVA gel polymer electrolytes, as shown in Figure 4h, where the Ti_3_C_2_T*_x_* @Zn anode acts as an ion sieving layer which prevents dendrite formation, suppresses HER, and limits passivation. The fabricated flexible Zn‐air battery delivers superior voltage stability for 200 cycles at 10 mA cm^−2^ current density compared to the bare Zn anode (Figure 4i). Besides, the flexible Zn‐air battery was tested at different bending angles from 0° to 180° where the charge–discharge curves remained unchanged, indicating better performance of the flexible Zn‐air battery (Figure 4j).

Adopting the MXene‐Zn model, Li et al. [64] designed the MXene‐Zn electrode with Cu‐modified Ti_3_C_2_Cl_2_ (Cu‐MXene) with high hydrophobic and zincophilic properties, which serve as a protective coating on Zn anode and provide mass Zn nucleation sites with uniform charge distribution, leading to uniform Zn deposition (Figure 4k). The Cu‐MXene‐Zn exhibits a relatively lower OCV compared to bare Zn and MXene‐Zn anode (Figure 4l). Besides, the fabricated Zn‐air battery with Cu‐MXene‐Zn anode delivered higher durability for 1000 h at 10 mA cm^−2^ current density and 1 mAh cm^−2^ specific capacity (Figure 4m). Notably, the MXene/Zn‐LDH‐array@PVA electrode, as shown in Figure 4n, shows a layered structure that allows feasible Zn deposition and suppressed HER [65]. The MXene/Zn‐LDH‐array@PVA delivers a high specific capacity of 645 mAh g_Zn_^−1^ at 3 mA cm^−2^ current density compared to Mix‐PVA@LDH and pure PVA (Figure 4o). The layered structure facilitates uniform Zn deposition during charge–discharge cycles, reducing the risk of dendrite formation.

**FIGURE 5:**
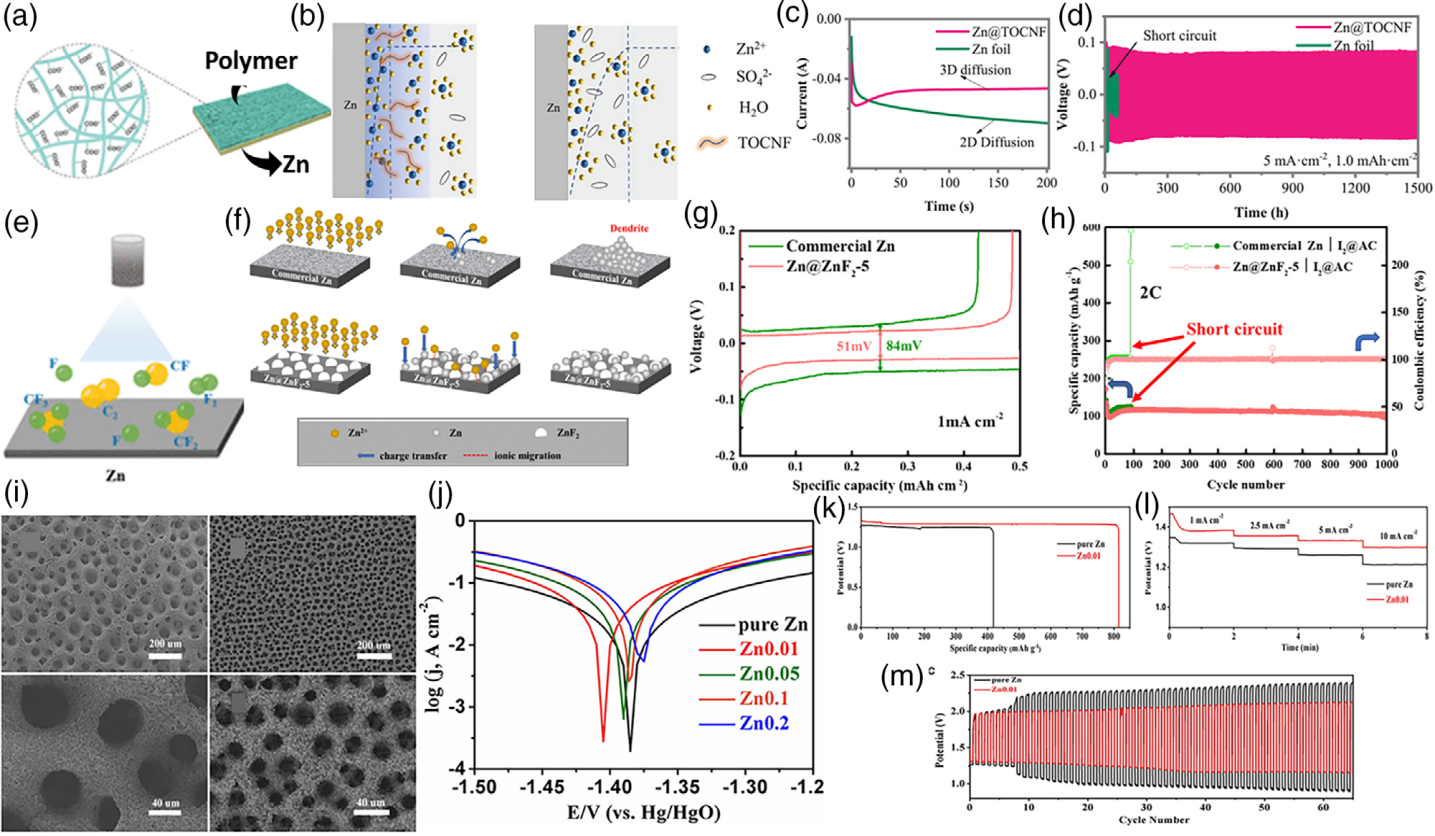
(a) Representation of Zn anode with polymer coating. (b) Illustration of ZAB performances with polymer coated Zn anode. (c) Chronoamperometry curves and (d) charge–discharge curves for polymer coated Zn anode, respectively. Reproduced with permission [66]. Copyright 2022, Wiley‐VCH. (e) Representation of F coating over Zn anode. (f) Illustration of Zn dendrite suppression over Zn@ZnF_2_ anode. (g) Polarization curves and (h) cycling stability of Zn@ZnF_2_ anode for ZAB performances. Reproduced with permission [67]. Copyright 2023, American Chemical Society. (i) SEM images of Zn anode with various pore sizes. (j) Polarization curves for ZAB with various Zn anode at different pore sizes. (k) Specific capacity curves. (l) Chronopotentiometry curves, and (m) charge–discharge curves for ZAB with Zn0.01 anode, respectively [68].

Other than MXene‐based Zn metal anodes, coating Zn metal anodes with polymer and non‐metals, like F and N, was also followed to minimize corrosion, dendrite formation, and solvation effects. Therefore, Li et al. [66] designed the Zn anode with a polymer coating made of TEMPO‐oxidized cellulose nanofiber (TOCNF), which helps to buffer the huge volume variation (Figure 5a). As shown in Figure 5b, the electrode–electrolyte interface for Zn@TONCF suggests a uniform deposition of Zn ions during charging cycles due to better hydrophilicity than bare Zn anode. The hydrophilic TOCNF in the Zn anode enhances mass transfer, which exhibits stable 3D diffusion, as shown in Figure 5c. The Zn@TOCNF anode displays exceptional cycling stability over 1500 cycles at 5 mA cm^−2^ current density and 1 mAh cm^−2^ specific capacity compared to Zn foil (Figure 5d). However, the bare Zn foil undergoes short circuit under similar conditions, possibly due to dendrite formation.

Besides, coating the Zn anode with non‐metal elements is a promising strategy to enhance the battery capacity. Therefore, Li et al. [67] prepared the uniform ZnF_2_ particles using the CF_4_ plasma technology on commercial Zn (Figure 5e). Due to better ionic conductivity and poor electronic conductivity of ZnF_2_, ion and electron distribution is orderly regulated at the anode, which guides the uniform Zn deposition nature and limits dendrite growth (Figure 5f). Zn@ZnF_2_ exhibits a limited charge–discharge overpotential of about 51 mV compared to commercial Zn anode, as shown in Figure 5g, due to the suppression of dendrite formation and undesirable side reactions. The superior cycling stability and Coulombic efficiency for the Zn@ZnF_2_ anode show the effectiveness of the simple F coating over the Zn anode compared to the commercial Zn anode, which undergoes a short circuit after 100 cycles (Figure 5h).

Other than coating strategies, Liu et al. [68] designed the 3D porous network structure by tuning the pore sizes via electrodeposition (Figure 5i). As observed, Zn0.01 electrodes with lower pore sizes exhibit lower required OCV than samples with higher pore sizes, as shown in Figure 5j. The Zn0.01 electrode reveals a higher specific capacity of about 820 mAh g^−1^ due to the highly exposed electrode surface due to the smaller pore sizes (Figure 5k). Besides, the discharge curves at varied current densities reveal relatively higher voltage, indicating the effectiveness of smaller pore sizes at the Zn anode (Figure 5l). The cycling stability tests also reveal lower voltage differences and better cyclability for charge–discharge performances than the pure Zn anode (Figure 5m). Hence, a simple strategy of utilizing lower pore sizes at the Zn anode to achieve highly exposed sites and tuning the surface energy for Zn deposition enables higher Zn‐air battery performances than conventional Zn anode designs.

The Zn anode can be prepared as alloy composites to limit dendrite formation and enhance its corrosion resistance. Notably, the Zn‐Sn alloy prepared by Peng et al. [69] suggests limited dendrite formation and better corrosion resistance. The charge–discharge performances of the Zn‐Sn alloys deliver stable performances and lower charge–discharge voltage differences compared to bare Zn anode. The fabricated Zn‐air battery delivers a high‐power density of 100 mW cm^−2^ and durable performances for 200 h. Besides, Lee et al. [70] prepared the Zn‐Ni‐In alloys at a 90:7.5:2.5 ratio which delivers a higher current response for Zn‐air battery compared to pure Zn anode. The SEM analysis after cycling tests reveals uniform Zn deposition, higher HER overpotential, and limited corrosion of the Zn anode for Zn‐Ni‐In alloys. Hence, using Zn‐based alloys as the anode proves to be a promising strategy to improve the Zn‐air battery performance.

## 5 Electrolyte Modification

Electrolyte modification plays a vital role in stabilizing zinc anodes in zinc‐air batteries. Conventional carbonate‐ or phosphate‐based electrolytes often cause corrosion and degradation of zinc anodes, reducing battery life and performance. To address this issue, researchers developed alternative electrolyte systems similar to lithium‐ion batteries, which exhibit improved robustness and durability. Furthermore, non‐aqueous electrolytes, such as organic acids, can enhance permeability and thermal stability. By designing an electrolyte system, researchers aim to improve zinc‐air batteries' overall performance and lifetime. The electrolyte content determines the concentration of the zincate ions and the redox reactions at the electrode surface. The unwanted side reaction at the anode is HER, which occurs at a relatively lower potential of −0.83 V_SHE_ than is required for Zn/ZnO (−1.26 V_SHE_) [60]. Therefore, using polymers, ionic liquid, and iodine redox‐based electrolytes and modified electrolyte concentration inhibits HER [25, 71]. Besides, the zincate ions are converted to solid ZnO, which at supersaturation is deposited densely at the Zn electrode, inhibiting the reversibility of the Zn‐air battery. Hence, tuning the electrolyte with appropriate concentration and inclusion of additives effectively suppresses the undesired by‐products during the charge–discharge cycles of the Zn‐air battery.

In the case of aqueous electrolytes, the electrolyte degradation effects are minimal however, HER is inevitable due to higher concentration of protons in water [25]. In non‐aqueous electrolytes like ionic liquids, HER can be significantly minimized, but electrolyte degradation at higher potentials becomes a major concern [25]. For example, Alwast et al. [72] studied the electrolyte decomposition effects using differential electron mass spectrometry (DEMS) with ionic liquid *N*‐butyl‐*N*‐methyl pyrrolidinium bis(trifluoromethanesulfonyl)‐imide (BMP‐TFSI). The DEMS study in non‐aqueous ionic electrolytes also reveals H_2_ evolution sourced from the BMP‐TFSI decomposition fragment [73]. Hence, the electrolyte decomposition in aqueous conditions may lead to HER, and in the case of non‐aqueous electrolytes, stability is compromised, affecting the specific capacity of the Zn‐air battery.

The non‐aqueous electrolytes may corrode the components of the battery over long cycles, which reduces its efficiency and life span. The thermal instability of the non‐aqueous electrolytes leads to electrolyte decomposition, reducing the performance of the Zn‐air battery. Besides, non‐aqueous electrolytes, such as ionic liquids, enhance the ionic conductivity which affects the power output efficiency of the Zn‐air battery compared to aqueous electrolytes. Notably, during discharge, the water molecules are created within the non‐aqueous conditions which is highly challenging to maintain. Non‐aqueous electrolytes are expensive, flammable, toxic, and create safety concerns for environmental conditions [74]. Hence, non‐aqueous electrolytes present critical limitations over aqueous electrolytes for Zn‐air batteries.

**FIGURE 6:**
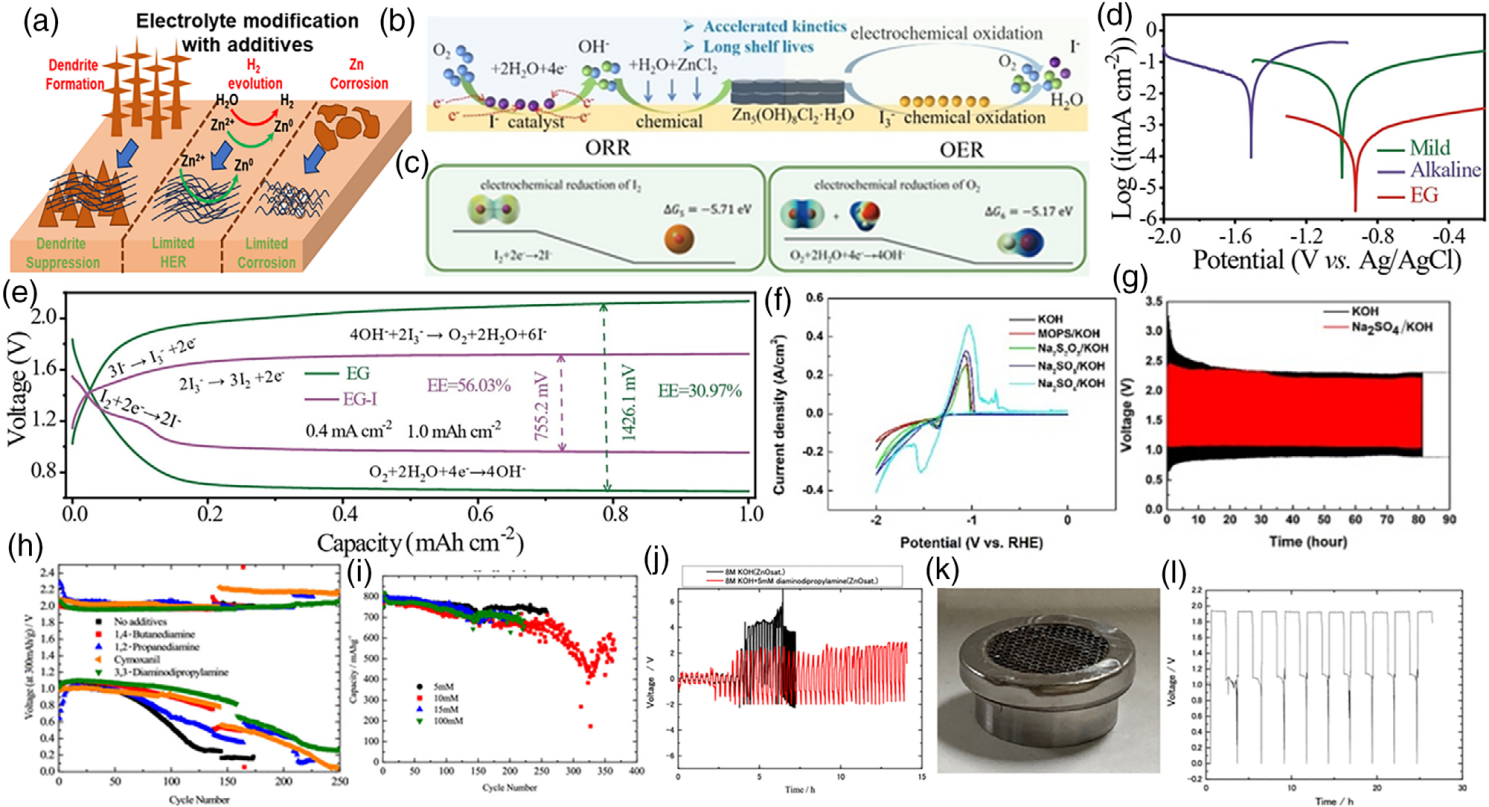
(a) Scheme for electrolyte modification to suppress dendrite formation. (b) Mechanism of ORR and OER supported by I_3_^−^/I^−^ redox reactions with electrolyte additives. (c) Charge density plots for ORR and OER supported by I_3_^−^/I^−^ redox reactions with electrolyte additives. (d) Polarization curves for ZAB with various electrolytes. (e) Potentiometric curves for ZAB with I_3_^−^/I^−^ redox kinetics in ethylene glycol (EG) electrolyte. Reproduced with permission [75]. Copyright 2023, Wiley‐VCH. (f) Polarization curves and (g) charge–discharge curves for ZAB with various inorganic additives in alkaline electrolytes, respectively. Reproduced with permission [76]. Copyright 2023, Elsevier. (h) Charge–discharge curves for ZAB with various organic additives in ZAB. (i) Discharge curves with 3,3‐diaminodipropylamine at various concentrations. (j) Charge–discharge curves with 5 mM 3,3‐diaminodipropylamine additive in alkaline electrolyte. (k) Optical image and (l) charge‐discharge curves of ZAB with organic electrolyte. Reproduced with permission [77]. Copyright 2023, American Chemical Society.

Notably, the additive in alkaline electrolyte effectively suppresses the Zn dendrite formation, limits HER, and reduces Zn anode corrosion (Figure 6a).

Cui et al. [75] designed the Zn‐air battery with accelerated kinetics regulated by I_3_^−^/I^−^ redox reactions (Figure 6b). The charging process by oxidation of Zn_5_(OH_8_)Cl_2_.H_2_O is mediated by I_3_^−^ oxidation and the discharging process by I^−^ adsorbed on catalyst accelerating ORR kinetics (Figure 6c). The Zn‐air battery under ethylene glycol (EG) electrolyte reveals a high electrochemical performance (Figure 6d). Besides, the EG electrolyte with an I‐based additive exhibits lower charge–discharge overpotentials of about 755.2 mV, indicating superior activity of the I_3_^−^/I^−^ redox reactions (Figure 6e). Similarly, Hosseini et al. [76] designed the Zn‐air battery electrolyte with four additives containing S–O anion groups, such as 4‐morpholinepropanesulfonic acid (MOPS, organic) and Na_2_S_2_O_3_, Na_2_SO_3_, and Na_2_SO_4_ (inorganic) to ensure good Zn‐deposition/dissolution (Figure 6f).

The 6 M KOH electrolyte containing 0.33% (v/v) 1 M Na_2_SO_4_ additive exhibits higher electrochemical activity and lower charge–discharge overpotentials with better Zn deposition, reducing the Zn dendrite formation (Figure 6g). Besides, Ishihara et al. [77] designed the Zn‐air battery electrolyte with organic additives, which critically affects the solvation effects of Zn metal, inducing better Zn deposition and dissolution (Figure 6h). Among the reported additives, 3,3‐diaminodipropylamine exhibits strong interaction with solvated water, which reveals enhanced electrochemical performances. Besides, the molar optimization of 3,3‐diaminodipropylamine shows 5 mM is the optimal concentration compared to other values, as shown in Figure 6i, and the charge–discharge performances with 5 mM 3,3‐diaminodipropylamine + 8 M KOH electrolyte exhibits strong cycle stability compared to the electrolyte without additive (Figure 6j). Besides, the coin cell performances were studied, and stable cycling performances were observed without degradation (Figure 6k,l).

**FIGURE 7:**
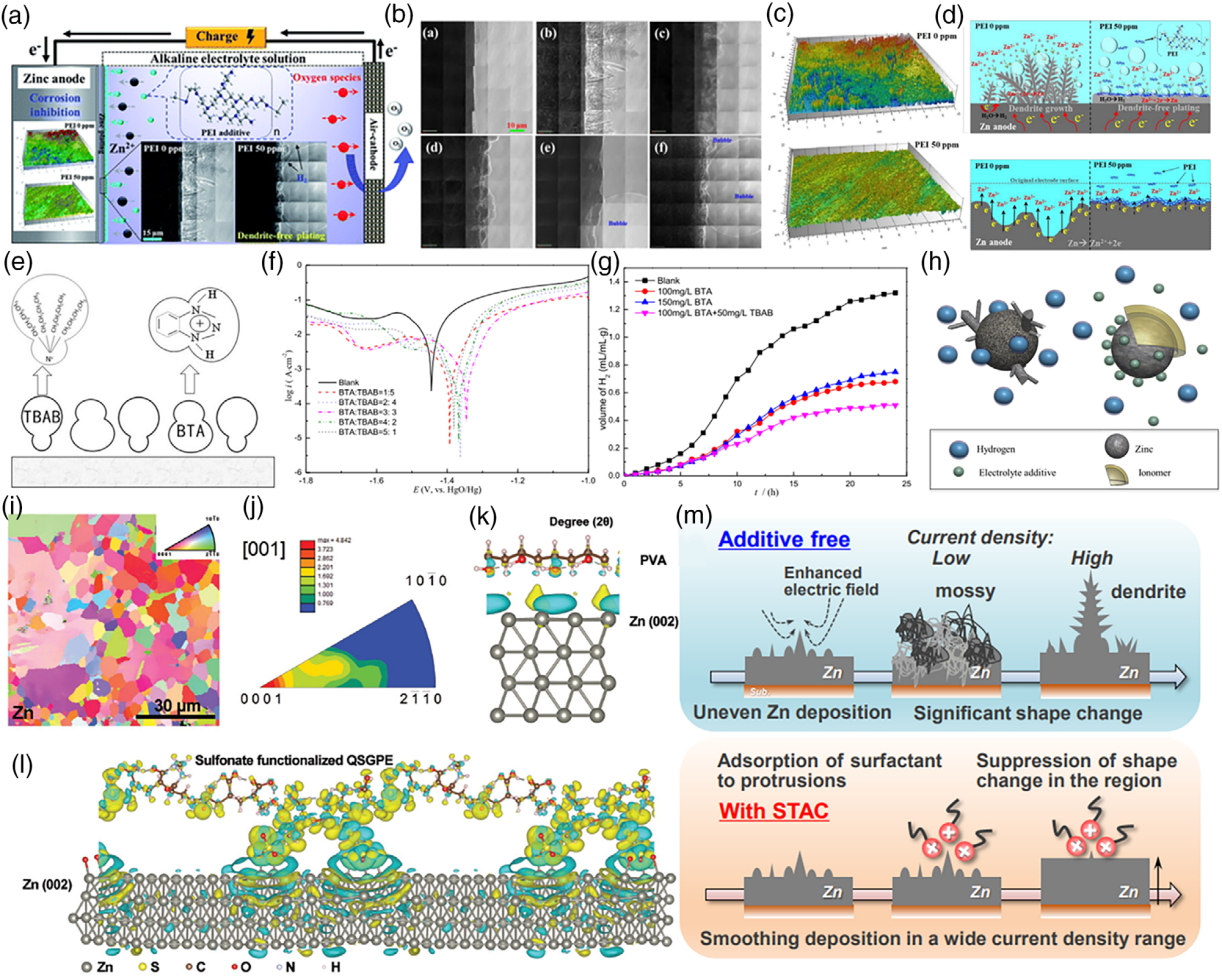
(a) Representation of alkaline ZAB with organic additives, (b) in situ TXM images, (c) in situ SEM images of ZAB with organic electrolyte, respectively, and (d) representation of dendrite suppression with organic additives. Reproduced with permission [78]. Copyright 2020, Royal Society of Chemistry. (e) Representation of TBAB and BTA electrolyte in ZAB anode, (f) polarization curves, and (g) H_2_ evolution curves with TBAB and BTA additives in alkaline ZAB Reproduced with permission [79]. Copyright 2020, Springer Nature. (h) Representation of dendrite suppression in Zn anode [80]. EBSD images representing: (i) orientation mapping images and (j) inverse pole figures of Zn anode, and charge density figures (k) without and (l) with SFQ electrolyte in flexible ZAB, respectively. Reproduced with permission [81]. Copyright 2023, Wiley‐VCH. (m) Representation of dendrite suppression with and without STAC additive in alkaline ZAB Reproduced with permission [82]. Copyright 2023, Royal Society of Chemistry.

Lin et al. [78] designed the alkaline electrolyte with polyethyleneimine (PEI) additive to restrict the dendrite formation and inhibit corrosion (Figure 7a). The in situ transmission X‐ray microscopy (TXM) analysis reveals uniform Zn deposition as the PEI concentration increases (Figure 7b).

The addition of PEI also limits the self‐corrosion and limits HER over the anode, which reveals a high Zn corrosion inhibition efficiency of about 52.2% with 50 ppm PEI, as shown in Figure 7c, and the surface roughness is significantly reduced (Figure 7d). Similarly, Wang et al. [79] utilized benzotriazole (BTA) and tetrabutylammonium bromide (TBAB) as additives, which effectively inhibit HER and corrosion‐suppressing dendrites and delay passivation (Figure 7e,f). The Zn‐air battery exhibited higher electrochemical performances with lower HER at optimal 100 and 200 mg L^−1^ of BTA and TBAB, respectively (Figure 7g). The ionomers were coated as a protective layer for dendrite formation, which limits HER and promotes the Zn‐air battery performance (Figure 7h) [80].

Besides, Fan et al. [81] designed the gel polymer electrolyte with sulfonate functionalized Zn‐air battery quasi‐solid‐state gel polymer electrolytes (QSGPE) for alkaline tolerance and Zn anode stability in flexible Zn‐air batteries. The electron backscatter diffraction (EBSD) results of the used Zn anode with sulfonate functionalized nanocomposite QSGPE (SFQ) for orientation mapping images (Figure 7i) and inverse pole figure (Figure 7j) reveal the minimal roughness of the Zn anode after cycling. The DFT analysis with SFQ estimates the strong interaction of SFQ groups with the Zn anode, stabilizing the dendrite formation (Figure 7k,l). Figure 7m reveals the significance of trimethyloctadecylammonium chloride (STAC) additives in alkaline electrolytes to limit dendrite suppression [82]. Table 1 compares previously reported research on Zn‐air batteries to help readers better understand the recent trends in the field.

**TABLE 1:** Comparison of performances of previously reported Zn‐air batteries.

Cathode	Anode	Electrolyte	Strategy	Specific Capacity (mAh g^−1^)	Power density (mW cm^−2^)	Polarization gap (V)	Coulombic efficiency	Ref.
Zn anode modifications								
Pt/C+IrO_2_	Ti_3_C_2_T*_x_* @Zn	6 M KOH and 0.2 M Zn(AC)_2_	MXene/Zn metal interfacial design	—	—	0.63	—	[63]
NaV_3_O_8_·1.5H_2_O	Cu‐modified Ti_3_C_2_Cl_2_/Zn	ZnSO_4_ (2 m)	Protective MXene coating coating on Zn anode	426	—	0.366	99.6%	[83]
Pt/C + RuO_2_/CC	MXene/Zn‐LDH‐array@ PVA	PVA	Electrode–electrolyte integrated MXene/Zn‐LDH array@ PVA structure	640.3	92.3	—	—	[65]
δ‐MnO_2_	Zn@TOCNF	2 M ZnSO_4_	Protective coating of TEMPO‐oxidized cellulose nanofiber over Zn	100	—	—	∼100%	[66]
Activated carbon	Zn@ZnF_2_‐5	2 M ZnSO_4_	Zn anode modification with uniform nanoscale ZnF_2_ particles	10 mA h cm^−2^	—	0.072	97.6%	[67]
Pt/C	Porous Zn	6 M KOH	Porous Zn with three‐dimensional (3D) network frame structure	812	—	1.33 V	0.63 V	[68]
Electrolyte modifications								
S‐C_3_N_4_ and CNS	Zn foil	Zn_5_(OH)_8_Cl_2_⋅H_2_O with I_2_ (0.025 M)	Accelerated kinetics mediated by I_3_^−^/I^−^ redox	—	—	0.755	Energy efficiency: 56.3%	[75]
Co_3_O_4_‐NC/CNT	Zn foil	4‐Morpholine propane sulfonic acid/0.33% (v/v) 1 M Na_2_SO_4_/6 M KOH	Additives having an S‐O anion group offer varied charge distributions	818.31 (50 mA cm^−2^)	216.16	—	—	[76]
Ni_0.8_Fe_0.2_ Co_2_O_4_	Zn foil	3,3‐diamino dipropyl amine/8 M KOH‐H_2_O	Strong interaction between 3,3‐diamino dipropylamine and solvated water	800	—	—	—	[77]
Pt/C and IrO_2_	Zn foil	6 M KOH/ 0.3 M ZnO‐50 ppm PEI	Deposited Zn nuclei are changed from a spiky dendritic structure to a dense film with a PEI concentration	—	—	—	Corrosion inhibition efficiency: 52.2%	[78]
Nano‐La_0.7_Sr_0.3_ Mn_0.6_Ni_0.4_O_3_	Zi–Bi powder	6 M KOH ‐ 100 mg L^−1^ BTA + 50 mg L^−1^ TBAB	Inhibition of HER and dendrite formation with organic additives	475.6	—	—	—	[79]
α‐MnO_2_‐CNT	Zn powder with In and Bi	4 M KOH/ ZnO, KF, K_2_CO_3_	Electrolyte additives and ionomer coating regulate the exposition of Zn	666	Energy density: 25 Wh kg^−1^_Zn_	—	—	[80]
—	Zn foil	PVA–KOH QSGPE	Quasi‐solid‐state gel polymer electrolytes (QSGPEs)	242.23 mAh cm^−3^	104.2 mW cm^−2^	—	—	[81]
Cu substrate	Zn plate	0.25 M ZnO + 4 M KOH with 1 mM SDS/PAA	Surfactants as an inhibitor of the formation of mossy and dendrite Zn	—	—	—	95%	[82]

Notably, flexible Zn‐air batteries in 1D and 2D structures have been studied and developed and are anticipated to levitate the research grounds toward more futuristic technologies. Flexible batteries offer compaction and more manageable transport, which can revolutionize the digital era by developing energy storage devices that can fit anywhere, allowing more space to occupy. Table 2 summarizes some recently reported flexible Zn‐air battery devices in 1D and 2D structures, which can be visualized as threads, rods, fabric, sheets, etc. Hence, the development and advancements of Zn‐air batteries have shown that efficient energy storage technologies can empower digital mankind with endless capabilities and promote the transition to green energy to achieve sustainability.

**TABLE 2:** Comparison of electrolytes and different Zn anode for flexible Zn‐air battery.

Electrolyte	Anode	Cathode	Energy density (Wh kg^−1^)	Power density (mW cm^−2^)	Reference
PAM	Zn foil	MnO_2_/NRGO	—	105	[84]
Porous PVA + SiO_2_	Zn plate	Co_3_O_4_	—	80.9	[85]
Porous PVA	Zn powder	Co_3_O_4_+ LaNiO_3_/ NCNT	581	—	[86]
Functionalized biocellulose	Zn foil	Fe‐HIB‐MOF@SS	975	194	[87]
PVP	Zn powder	FeNC‐1	—	250	[88]
PVA	Zn deposition @Cu film	Ultrathin Co_3_O_4_	546	—	[89]
PANa‐cellulose	Zn@ CNTP	Fe‐N‐C@CNTP	930	210.5	[90]
Acrylic polymers	Zn fiber	NC‐Co/CoN_x_	—	104	[91]
PVA@Chiffon band	Zn wire	Co_3_O_4_/N‐rGO	649	—	[92]
Gelation	Spiral Zn foil	Fe/N/C	—	—	[93]

## 6 Conclusion

In summary, the zinc‐air batteries use oxygen as the cathode and zinc metal as the anode. The battery has a high energy density and long lifespan, which makes it ideal for electric vehicles. Zinc anodes have many benefits, but their design critically affects their performance. The performance of Zn‐air batteries is affected by dendrite formation, hydrogen evolution reaction (HER), and solvation effects. The issues in the Zn anodes are addressed by engineers using hybrid composite materials, protective coatings, and various additives in alkaline electrolytes. Improved anode design is essential to the advancement of this technology. Zn anode stabilization is the key to improving the battery's efficiency, long‐term endurance, and use as an energy storage device. In addition, the review suggests a few perspectives towards the development of Zn‐air battery technology which can offer better research orientation and rapid commercialization of Zn‐air battery devices.

1. Aqueous Zn‐air batteries are highly promising compared to non‐aqueous Zn‐air batteries due to their feasibility of handling, low cost, and environmental compatibility. However, to few inimical factors like dendrite formation, corrosion, HER, surface passivation pose a disadvantage to the Zn anode stabilization. The instability of the Zn anode greatly reduces the battery performance and rechargeability. Therefore, researchers have proposed the development of Zn anode designs like Zn@support, Zn@polymer, non‐metal doped Zn, and Zn‐based alloys that can help enhance the stability of anode during charge–discharge cycles.
2. Electrolytes play a crucial role in the stabilization of the Zn anode since electrolytes control ionic transport and determine the dissolution/deposition of Zn ions during charge–discharge cycles. Aqueous electrolytes with H_2_O act as a major source of protons which facilitates HER inevitably and allows for dendrite formation on the Zn anode. Researchers have followed several electrolyte modifications by introducing additives, like the inclusion of surfactants, metal salts, and oxides to inhibit the effects of Zn corrosion and ensure uniform Zn deposition. Besides, using sulfonate‐based ionic liquids has also proven to be an effective method to improve corrosion resistance, inhibit dendrite formation, suppress HER, and avoid electrode passivation during cycling tests.
3. Several theoretical and computational studies have provided an insightful understanding of the Zn anode stabilization and significant methods to solve the issues faced during charge–discharge cycles. Notably, machine learning methods can be applied to the aqueous Zn‐air battery systems to obtain an efficient choice of elements and strategies for Zn anode design. Similarly, the choice of electrolyte and its additives can be screened in a controlled environment to achieve highly efficient real‐time Zn‐air battery designs hassle‐free. Researchers have utilized machine learning‐based quantitative structure‐property relationship (QSPR) methods to demonstrate the corrosion resistance property of various organic compounds and allow the choice of appropriate electrolyte additives without much information about the corrosion‐resistant mechanism.
4. In situ and ex situ characterization techniques during and after battery cycling tests provide deeper knowledge about the undergoing reactions and mechanisms at the electrode–electrolyte interface. This allows the researchers to adopt an efficient method by knowing the exact cause of performance degradation of the Zn‐air battery.
5. The concept of flexible batteries is anticipated to be the future of all energy storage technologies which allows for compaction and easier transport. Therefore, the research on flexible Zn‐air batteries had several major advancements and still requires more study before commercialization. Batteries that can be deformed several times and still retain their performances are highly anticipated which provides a futuristic approach towards energy storage devices.

## Conflicts of Interest

The authors declare no conflicts of interest.
